# Analytical and Clinical Validation of Assays for Volumetric Absorptive Microsampling (VAMS) of Drugs in Different Blood Matrices: A Literature Review

**DOI:** 10.3390/molecules28166046

**Published:** 2023-08-14

**Authors:** Rhea Veda Nugraha, Vycke Yunivita, Prayudi Santoso, Aliya Nur Hasanah, Rob E. Aarnoutse, Rovina Ruslami

**Affiliations:** 1 Doctoral Study Program, Faculty of Medicine, Universitas Padjadjaran, Bandung 40161, Indonesia; rhea19002@mail.unpad.ac.id; 2 Division of Pharmacology and Therapy, Department of Biomedical Sciences, Faculty of Medicine, Universitas Padjadjaran, Bandung 40161, Indonesia; v.yunivita@unpad.ac.id; 3 Division of Respirology and Critical Care, Department of Internal Medicine, Faculty of Medicine, Universitas Padjadjaran—Hasan Sadikin Hospital, Bandung 40161, Indonesia; prayudimartha@yahoo.com; 4 Department of Pharmaceutical Analysis and Medicinal Chemistry, Faculty of Pharmacy, Universitas Padjadjaran, Bandung 45363, Indonesia; aliya.n.hasanah@unpad.ac.id; 5 Department of Pharmacy, Radboud University Medical Center, Research Institute for Medical Innovation, 6255 HB Nijmegen, The Netherlands; Rob.Aarnoutse@radboudumc.nl

**Keywords:** optimization, analytical validation, clinical validation, volumetric absorptive microsampling (VAMS), conventional venous sampling (CVS)

## Abstract

Volumetric absorptive microsampling (VAMS) is the newest and most promising sample-collection technique for quantitatively analyzing drugs, especially for routine therapeutic drug monitoring (TDM) and pharmacokinetic studies. This technique uses an absorbent white tip to absorb a fixed volume of a sample (10–50 µL) within a few seconds (2–4 s), is more flexible, practical, and more straightforward to be applied in the field, and is probably more cost-effective than conventional venous sampling (CVS). After optimization and validation of an analytical method of a drug taken by VAMS, a clinical validation study is needed to show that the results by VAMS can substitute what is gained from CVS and to justify implementation in routine practice. This narrative review aimed to assess and present studies about optimization and analytical validation of assays for drugs taken by VAMS, considering their physicochemical drug properties, extraction conditions, validation results, and studies on clinical validation of VAMS compared to CVS. The review revealed that the bio-analysis of many drugs taken with the VAMS technique was optimized and validated. However, only a few clinical validation studies have been performed so far. All drugs that underwent a clinical validation study demonstrated good agreement between the two techniques (VAMS and CVS), but only by Bland–Altman analysis. Only for tacrolimus and mycophenolic acid were three measurements of agreement evaluated. Therefore, VAMS can be considered an alternative to CVS in routine practice, especially for tacrolimus and mycophenolic acid. Still, more extensive clinical validation studies need to be performed for other drugs.

## 1. Introduction

The essential goal of clinical pharmacology is to understand the dose–concentration–effect relationship. The study of pharmacokinetics seeks to explain the time course of drug concentrations in the body (dose–concentration relationship). However, the time course of drug concentrations cannot predict the magnitude of the drug effect (concentration–effect relationship) [1,2]. This dose–concentration–effect relationship is now used as the central concept of therapeutic drug monitoring (TDM), which individualizes drug dosage by attaining specific target plasma concentrations (therapeutic ranges) guided by the measurement of plasma drug concentrations [3]. TDM is used for assessing efficacy, diagnosing undertreatment, preventing adverse effects, guidance to stop the treatment, monitoring and detecting drug interaction, and monitoring adherence [4]. It is recommended for drugs with a narrow therapeutic range, significant pharmacokinetic variability, and a clear relationship between drug concentrations and clinical response [5]. 

The sampling technique commonly used to routinely measure drug concentration, either for TDM or pharmacokinetic studies, is conventional venous sampling (CVS), which draws blood from the vein. CVS can lead to unexpected complications, such as hematoma and thrombophlebitis [6,7]. This technique requires much blood for each sample (up to 5 mL), is invasive and inconvenient, has a relatively high cost, sometimes needs special conditions (a cold chain) for transport, and samples must be centrifuged before storage as plasma or serum. If the sample is not immediately analyzed, it must be frozen at −20 up to −80 °C. These characteristics cause CVS to be less flexible for application in the field [8,9,10]. An advantage of CVS is that the sample volume is large, while analysis only needs a small volume; therefore, the leftover sample could be stored and re-analyzed [6,7].

Considering the many limitations of CVS, which can complicate TDM programs or pharmacokinetic studies, there have been many innovations in sampling techniques for drug concentration measurement in the past decade, called microsampling techniques. Microsampling is a sampling technique that only takes a small volume of samples (<100 µL for blood) from the body for analysis; it is less invasive, less painful, and more efficient. This technique is also more practical compared to CVS [11]. Therefore, this technique may improve routine clinical care in TDM and benefit pharmacokinetic studies, including those with children as subjects. Some microsampling techniques that have already been developed are capillary microsampling (CMS), dried blood spots (DBS), dried plasma spots (DPS), plasma preparation technologies, solid-phase microextraction (SPME), and volumetric absorptive microsampling (VAMS) [12]. VAMS and DBS have been the most researched to analyze drugs [12,13,14,15,16,17]. Previously, the most commonly used and straightforward microsampling design was DBS, which requires small volume sizes (30 µL per spot). But DBS has some limitations primarily related to the effect of hematocrit values that may affect measured concentrations. The VAMS technique was then developed to tide over the boundaries of DBS [12,18,19]. VAMS is a promising sample-collection technique for the quantitative analysis of drugs, which only takes a 10–50 µL volume of samples by an absorbent tip [20,21]. Compared to CVS, the VAMS technique has some superiority in practicality and flexibility of the sampling process because it only needs a small sample volume. The sampling process is relatively fast and less invasive and there is no need to freeze the sample before transport; thus, it may be more cost-effective, there is no need for centrifugation before storage, and it also reduces the amount of freezers needed to keep samples in the long term [18,19,22]. Therefore, this VAMS technique is more flexible for application in the field than CVS and can be innovative for sampling processes that are more practical and simpler [18,22].

Analytical methods for drugs need to be optimized and validated before the method is used [23]. Furthermore, VAMS can only be implemented in routine practice care, replacing CVS, after it has been successfully validated in a clinical validation study. In a clinical validation study, paired drug concentrations from samples taken by VAMS and CVS are obtained from actual patients and are statistically analyzed [24]. Until now, no review has described the clinical validation study of drugs that have undergone optimization and analytical validation. But there was a review describing the analytical validation of some drugs [25].

This narrative review aimed to assess and present studies about the optimization and validation of assays for drugs taken by VAMS, considering their physicochemical properties, extraction conditions, validation results, and the clinical validation of VAMS versus CVS.

## 2. Volumetric Absorptive Microsampling (VAMS)

**Figure 1:**
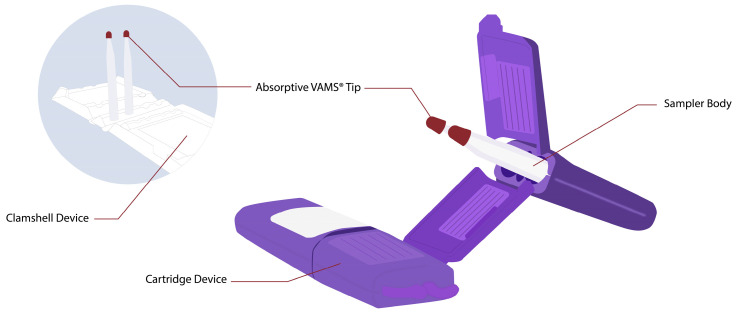
Volumetric absorptive microsampling devices (**left** picture is VAMS in clamshell device; **right** picture is VAMS in cartridge device).

VAMS is the newest innovation in microsampling techniques. VAMS was designed to absorb a minimal and fixed volume, such as 10, 20, and 50 µL. VAMS devices contain a white hydrophilic pore absorbent tip attached to a plastic handler [19]. The picture of the VAMS device is shown in Figure 1, which comes with two different types of devices, namely clamshells and cartridges. The sampling procedure with VAMS is simple: dipping the tip of VAMS in the location that has been punctured using a lancet at a 45° angle for 2–4 s until the tip is entirely red. The sample for this device is not only blood; VAMS has also been used to collect urine, saliva, or other liquid biological samples [18,19]. After obtaining the sample, the devices are dried at room temperature for at least 2 h. The samples can be transferred at room temperature for storage or analysis [19,26]. The drying of the devices could correlate with the limitation of VAMS itself. Care is also taken to ensure that the tips do not touch either other tips or their surroundings to prevent blood transfer during drying. If it touches others, it could be contaminated. Also, the variation in drying times could be an issue with reproducible recovery. Drug extraction is more inconvenient when samples become drier. Understanding the effect of drying on VAMS samples is essential for method development and validation. The total time for drying depends on the size of the absorbent tip on the VAMS devices. It has been known that 1 h of drying at room temperature is adequate when using 10 µL VAMS. Furthermore, the drying time also could generate the degradation of the sample; thus, the stability of analytics in drying time needs to be evaluated [18,27]. 

The dried sample from the VAMS technique has better stability than a liquid sample. Some studies reported that the desired analyte was stable for over a month in dried conditions at room temperature for an extended period (more than one month) [28,29,30,31,32,33,34,35,36,37]. Storage conditions need to be considered for the samples obtained from the VAMS technique, such as storing them in a closed and dark container and having a desiccant to prevent degradation [22]. The VAMS manufacturer provides clamshell storage with a hole for airflow that is important for drying. If the appropriate storage conditions are applied, it could extend the stability of the drugs [16,22,38]. However, stability still depends on many factors, such as analyte and storage conditions (temperature and how to save the sample). In the VAMS device, samples need to be stored in a tightly closed container that is dark and possibly with desiccant. Therefore, stability parameters must be evaluated during analytical method validation [22]. 

For the sample analysis, sample preparation must be conducted for the extraction process. Before the extraction, the tip from VAMS devices should be released from its handler [13,19,22,26,28], or the whole VAMS device could be used using automatic machines [19,22,39] to overcome the hematocrit issue of DBS [19]. The choice of type and solvent volume, extraction time, and extraction procedure are the most important in sample preparation for the VAMS technique. Ideally, a suitable extraction procedure is a procedure that can result in high reproducibility, maximal recovery of drugs, and minimal matrix effect [19,40]. In studies that used the VAMS technique, the type of solvent varied from 100% water [30,38,41,42,43] to 100% organic solvent [13,26,31,32,39,44,45,46,47,48]. Acid or base could be added to increase recovery [13,28,40,42,43,46,49], and the extraction time varied from minutes to one hour. A longer extraction time could increase the analyte’s recovery [19,39]. The VAMS technique, since it is a microsampling device, has a sensitivity issue based on its volume. It is known that the reduced sample volume impacts the sensitivity since the smaller volume also leads to small analyte/concentrations. Nevertheless, the small blood volume is sufficient for clinical assays because modern instruments are highly sensitive and can detect low concentrations of drugs and metabolites. Consequently, this highly sensitive instrument could overcome the issue of sensitivity of small-volume samples, especially in VAMS [27,50]. 

Assays for many drugs taken by VAMS have been optimized and analytically validated, such as cefepime, midazolam, tacrolimus, paracetamol, and voriconazole. Yet, only a few assays underwent clinical validations [26,51,52,53,54]. Some studies comparing VAMS and CVS demonstrated a strong correlation of drug concentrations from samples obtained by both sampling techniques, for example, lamotrigine, levetiracetam, phenytoin, valproic acid, and albendazole (r > 0.95) [51,52]. Furthermore, agreement analysis is also used for clinical validation. The clinical validation is discussed in another section [53,54].

## 3. Analytical Validation of Volumetric Absorptive Microsampling (VAMS) Assays of Drugs

### 3.1. Optimization and Validation

Before clinical validation, the analytical method of all drugs must be optimized and validated, which is different for every drug. Developing an analytical method consists of optimizing the method, including the extraction/detection of an analyte and subsequent validation of the analytical method [23]. Proper optimization and validation methods are essential for evaluating and interpreting bioavailability, bioequivalence, pharmacokinetic, and toxicokinetic research, which means it has a vital role in discovering, developing, and manufacturing drugs [55,56,57]. After optimization, the analytical method’s quality must be ensured to produce accurate and precise data [58,59]. Validation of the analytical method is a process to prove that the analytical method that has been optimized is appropriate for the analysis of the desired analyte [23]. Again, this process is vital to receive the quality and safety of the end product, especially in the pharmaceutical industry [58,59,60]. Selecting the extraction solvent and other conditions is essential for optimizing and validating. The extraction procedure should result in a reproducible, maximum recovery of the analytes of interest and low recovery of different compounds to reduce the matrix effect. The optimization of extraction conditions can be conducted by an experimental process [19,23].

#### 3.1.1. Optimization and Validation of Assays for Acidic Drugs

As can be seen in Table 1 for the optimization and validation for acidic drugs, the extraction solvent in the conducted studies with VAMS varied from water:methanol [61,62], methanol 100% [14,39,46,63], acetonitrile 100% [16,49], and methanol:acetonitrile [13,51]. Some studies added an acid or base to increase the recovery [13,46,51,62]. Vortexing was used in some studies [13,16,63]. Shaking [39,62], sonication [14,51,61,64], and vortexing–sonication were also used to optimize acidic drugs [46,63,64].

In addition, the extraction time varies between studies, from a few minutes to more than one hour. Increasing the extraction time may result in a higher recovery of the analytes [19]. A difference in the recovery of 8.8% was observed for tamoxifen between vortexing for one hour and a combination of vortexing for 1 min and sonication for 25 min (100.7% and 91.9%, respectively). But the use of different extraction solvents may also influence the result of their recovery [13,63]. Details can be seen in Table 1. 

Assays for acidic drugs, such as emixustat, paracetamol, cefepime, ethosuximide, felbamate, phenobarbital, phenytoin, primidone, topiramate, zonisamide, and tamoxifen were all validated and met the acceptance criteria by the FDA (Food and Drugs Administration) and/or EMA (European Medicines Agency) guidelines [16,46,62,63,64]. Other acidic drugs have also been validated, but some data were missing. Details of the validation results and the parameters missing from the analytical validation of acidic drugs are shown in Table 1, including the stability data. Most acidic drugs have a lower limit of quantification (LLOQ) below the concentration range in plasma or serum [65,66,67,68,69,70,71,72,73,74,75,76], except for atorvastatin with an LLOQ of 0.001 µg/mL [13], but clinically relevant concentrations are between 0.00033 and 0.01208 µg/mL [77]. Therefore, most acidic drugs could now undergo clinical validation, except atorvastatin, since its LLOQ was above the concentration range of the drug in plasma or serum.

#### 3.1.2. Optimization and Validation of Assays for Basic Drugs

The extraction solvent in the performed studies with VAMS for basic drugs varied from water 100% [38], water:methanol [28], water:acetonitrile [37], methanol 100% [14,31,45,78,79], acetonitrile 100% [16], methanol:acetonitrile [13,48,80], methanol:ethyl acetate [81], and methanol:zinc sulfate [82]. Some studies added an acid or base to increase the analyte’s recovery [13,28]. Vortexing is used in most studies [13,16,38,45,48]. Shaking [79], sonication [14,78], thermomixing [28,37], vortexing–sonication [80], thermomixing–vortexing [31], and sonication–shaking were also used in the optimization of basic drugs [81,82]. Details can be seen in Table 2. 

The use of extraction solvent, extraction time, and other conditions may influence the recovery of the analyte. However, there was no difference in the recovery of midazolam between using methanol 100% with a shaker for one hour and methanol–acetonitrile (1:1) with vortexing for one hour (99.95% vs. 101.2%) [13,79], which shows that the chosen combination of extraction solvent, extraction time, and other conditions may result in the same analyte’s recovery. 

Most basic drugs, including miltefosine, hydroxychloroquine, voriconazole, voriconazole N-oxide, lamotrigine, and rufinamide, have been validated analytically. Assays met the acceptance criteria by the FDA and/or EMA guidelines [16,31,38,80]. Also, other basic drugs described in Table 2 that have been validated showed some missing parameter data. Details of the validation results from the analytical validation of basic drugs are shown in Table 2, incorporating stability data and the analytical instrument used for each drug’s analytical method. The LLOQ of all basic drugs were below the concentration ranges of their drugs [13,14,16,28,31,37,38,45,48,70,78,79,80,81,82,83,84,85,86,87,88,89,90,91,92,93,94,95,96]. For example, mitotane has an LLOQ of 1 µg/mL [82], and its plasma concentration range is 11.3–23.3 µg/mL [92]. The assays for all other basic drugs could undergo clinical validation studies to compare with the assays after CVS.

#### 3.1.3. Optimization and Validation of Assays for Neutral Drugs

As can be seen in Table 3 for the optimization and validation of assays for neutral drugs, the extraction solvent in the conducted studies with VAMS varied from water:methanol [61], acetonitrile 100% [16], methanol:zinc sulfate [97], and acetonitrile:zinc sulfate [53]. No studies used acid or base modification to enhance recovery since the analyte is a neutral drug. The most used procedure was vortexing. It was used for carbamazepine, lacosamide, oxcarbazepine, perampanel, levetiracetam, and tacrolimus [16,53]. A shaker [97] was used for sonication for tacrolimus, sirolimus, everolimus, and cyclosporin A [61]. Three different conditions were used to extract tacrolimus. Shaking for 6 min [97], vortexing for 1 min [53], and sonication for 15 min [61] were used to extract tacrolimus, resulting in different recoveries (98.5%, 80%, and 81.8%, respectively). The use of a shaker to extract tacrolimus gives the best recovery. Still, the extraction solvent may influence the recovery since the three used different extraction solvents (methanol:zinc sulfate, acetonitrile:zinc sulfate, and water:methanol, respectively) [53,61,97].

All assays for neutral drugs, such as tacrolimus, carbamazepine, lacosamide, oxcarbazepine, perampanel, and levetiracetam, were validated and met the acceptance criteria by the FDA and/or EMA guidelines [16,97]. Table 3 shows the detailed validation results and the missing parameter data of other neutral drugs. Most neutral drugs that underwent a validation study have an LLOQ concentration below the plasma/serum concentration range of their drugs, except for everolimus, perampanel, and sirolimus [16,53,61,97,98,99,100,101,102,103,104,105,106]. Everolimus has an LLOQ of 0.02 µg/mL [61], whereas its concentration range is 0.003–0.008 µg/mL [100]. The LLOQ of perampanel was 0.022 µg/mL [16], and its concentration range was 0.019–2.436 µg/mL [104]. Sirolimus has 0.02 µg/mL as the LLOQ concentration [61] and a plasma/serum concentration range within 0.004–0.012 µg/mL [105]. Hereafter, most neutral drugs could undergo a clinical validation study since their LLOQ concentration was below the concentration range of their drugs, except for everolimus, perampanel, and sirolimus.

In summary, many drugs taken by VAMS underwent optimization and validation of analytical methods. Assays for acid, basic, and neutral drugs could be optimized and validated with different preparation or extraction procedures. Assays for most acidic drugs [16,18,44,51,52,65,66,67], most basic drugs [14,16,28,31,38,45,79,80], and all neutral drugs have been validated and met the acceptance criteria by FDA and/or EMA guidelines [16,53,61,97].

## 4. Clinical Validation of Volumetric Absorptive Microsampling (VAMS) of Drugs

Generally, a new sampling technique can only be implemented in routine practice by replacing CVS after it has been successfully validated in clinical validation. This clinical validation aims to show that the results from the new sampling technique are exchangeable with what is gained from the conventional one. In this case, a clinical validation study compares CVS with VAMS, obtained by finger prick, if both samples are taken simultaneously in the same patient (paired samples). The sample was analyzed and statistically evaluated. Ideally, the whole concentration range is validated based on a large enough sample size of at least 40 patients/subjects. The sample can be collected at a single time point (trough or peak), or paired samples are taken at 2–3 time points covering the whole concentration range in blood, serum, or plasma with for CVS volume samples according to standardized volume; for VAMS, this is adjusted to the tip used (10–50 µL) [24,107].

Clinical validation studies can incorporate three different agreement measures: Passing–Bablock/Deming regression analysis, Bland–Altman analysis displaying agreement, and an assessment of predictive performance [24,107]. Passing–Bablock/Deming regression analysis is used by plotting the concentration of the new VAMS technique against results from the conventional CVS technique as a standard concentration to allow for measurement errors on both the *x* and *y*-axis, and this technique is not hampered by a few outliers. Bland–Altman analysis is another means to provide insight into the agreement between the two techniques. The ultimate goal of VAMS blood concentration measurement is to predict the corresponding CVS (plasma) concentration. Predictive performance can be quantified by calculating the median percentage prediction error (MPPE), given by the median [100% × (Predicted concentration − Observed concentration)/Observed concentration]. Then, the median absolute percentage prediction error (MAPE) can be calculated by [100% × │(Predicted concentration − Observed concentration)│/Observed concentration]. The MMPE is a measure of bias, while the MAPE measures precision. Acceptance criteria can vary, but MMPE and MAPE <15% values are often applied [107]. Until now, only eight studies have performed a clinical validation study to compare VAMS and CVS, such as in albendazole, tacrolimus, radiprodil, and mycophenolic acid (Table 4). 

The analytical method for albendazole, a benzimidazole derivative and an anthelmintic for humans, from the sample taken by VAMS, was developed and validated by Schulz et al. [51] in 2019 and clinically validated by comparing the VAMS samples with plasma samples. In this study, 10 subjects receiving albendazole were sampled 10 times using CVS or VAMS for capillary blood until 24 h post-treatment. Bland–Altman analysis was used to determine the agreement between the two methods. The results showed a good agreement between the two matrices (VAMS blood and plasma). But in this study, measures of the agreement included only Bland–Altman analysis, and Passing–Bablock/Deming regression analysis and predictive performance were not assessed, and the number of subjects did not meet the minimum number of subjects [51]. Therefore, it would be better if the clinical validation of VAMS samples of albendazole were repeated using the three agreement measures with the minimum number of subjects in the future.

In 2020, Veenhof et al. [108] conducted a study about the clinical validation of VAMS and DBS versus CVS for tacrolimus. Tacrolimus is an immunosuppressant drug that has been part of routine transplant patient care for decades. This study included 88 matched samples from 72 patients, and VAMS and whole blood samples were taken during a regular visit. Because of insufficient sample quality, 62 duplicate VAMS samples were available for analysis. All three different measures of the agreement were performed. The Passing–Bablok fit was y = 0.88x + 0.01 (95% CI slope, 0.81–0.97; 95% CI intercept, −0.47–0.39), showing no significant constant difference, but a significant systematic difference of 12% lower tacrolimus concentration in VAMS to whole blood (WB) was established. This systematic difference was later used to obtain the following conversion formula: [tacrolimus WB concentration] = [tacrolimus VAMS concentration]/0.88. This conversion formula was used to recalculate all VAMS values, and these recalculated values were used in the Bland–Altman analysis. No significant bias was found in the Bland–Altman analysis, with a mean WB/VAMS ratio of 1.00 (95% CI 0.98–1.02), showing good agreement. Because of the correction factor, the bias estimation in the predictive performance was negligible. The MPPE and MAPE were within acceptable limits. This study suggested that VAMS (after correction) can be used in transplant patient care for tacrolimus monitoring [108].

Tacrolimus was also investigated by Vethe et al. [97] in 2019. In this study, two 12 h pharmacokinetic investigations were performed at the hospital in the early phase after transplantation (2–8 weeks after transplantation) when the tacrolimus dose was stable. Blood sampling, both VAMS and liquid venous blood, was performed at 13 time points after administration of individualized morning doses of tacrolimus. The agreement was measured using Passing–Bablock analysis, which was fit y = −0.5869 + 1.012x, and Bland–Altman analysis showed good agreement between the two matrices. It reported that less than 8% of the VAMS versus liquid venous sample pairs showed differences outside a ±20% range. Although the predictive performance was not assessed and the minimum number of subjects was not reached, the study suggested that the VAMS method is considered suitable for routine TDM in renal transplant recipients, either by trough or rich sampling strategies [97].

In 2020, an analytical method for immunosuppressant agents, such as mycophenolic acid, tacrolimus, sirolimus, everolimus, and cyclosporin, was developed and validated for samples taken by VAMS by Gonzalez et al. [61], and the two most used drugs (tacrolimus and mycophenolic acid) were clinically validated versus CVS/venous WB. In this study, 53 subjects (for tacrolimus) and 20 subjects (for mycophenolic acid) were obtained from adult patients on immunosuppressant treatment after liver transplantation. Passing–Bablock regression and Bland–Altman analysis were performed to evaluate the agreement between VAMS and CVS. Both tacrolimus and mycophenolic acid showed a strong linear relationship in the Passing–Bablock regression analysis. For tacrolimus, the association was not one of the equalities as the slope cannot be considered 1 since the estimated slope was 1.16, and its 95% CI (1.159–1.368) does not contain the value 1.0. Therefore, the estimated Passing–Bablok regression fit was used to transform VAMS concentration values into venous WB concentrations, and a new Passing–Bablok regression analysis was executed to assess the agreement between venous WB concentration and converted VAMS concentration. An agreement can be seen with an estimated slope of 0.997 (95% CI, 0.909–1.074). A robust linear relation and agreement can be seen for the mycophenolic acid. The estimated slope is similar to 1 (0.976 with 95% CI 0.936–1.053). Hence, no transformation or correction of the data is required. The MPPE and MAPE were within the acceptance criteria (<15%) for the predictive performance [61]. The VAMS method is suitable for routine TDM in liver transplant recipients for tacrolimus (after transformation/correction). For mycophenolic acid, more subjects should be added for another clinical validation before it can be used in routine practice. 

Zwart et al. [109] developed and validated an LC-MS/MS assay capable of quantifying tacrolimus, everolimus, sirolimus, cyclosporin, mycophenolic acid, creatinine, and iohexol simultaneously in DBS and VAMS samples. Tacrolimus, mycophenolic acid, creatinine, and iohexol assays were clinically validated against plasma in 2022. This study sampled twenty-five stable kidney transplant recipients after more than one year of receiving immunosuppressive therapy. Passing–Bablock regression showed adequate linearity to CVS. The Passing–Bablok regression slope was 1.05 (95% CI, 0.98–1.14) for tacrolimus for the individual concentrations. No correction was used for the Bland–Altman analysis. For mycophenolic acid, the Passing–Bablok regression slope of 0.72 (95% CI, 0.66–0.77); therefore, this data requires correction with VAMS to plasma conversion factors of 1/0.73, and after correction, the corrected VAMS mycophenolic acid concentrations showed adequate linearity with the plasma concentrations demonstrating a Passing–Bablok slope of 1.07 (95% CI, 0.97–1.13). Bland–Altman analysis showed good agreement [109]. Future research should add more subjects for further clinical validation of tacrolimus and mycophenolic acid. 

Radiprodil (UCB3491) is a selective negative allosteric modulator of NR2B-containing N-methyl-d-aspartate (NMDA) receptors and is currently under development for treating infantile spasms. An oral suspension was applied for pediatric use. In this study, radiprodil was involved in the clinical validation study in which 10 subjects were sampled at 15 time points post-dose, both with VAMS and CVS used for plasma samples. The agreement for this clinical validation was assessed using Bland–Altman analysis and showed good agreement with a mean bias of −11.4% (−39.1, 16.3). Passing–Bablock regression analysis and predictive performance assessment have yet to be performed [81]. We suggest that the other two agreement measures are assessed with a minimum of 40 subjects in future research. 

The results of clinical validation studies of several drugs are summarized in Table 4.

According to Table 4, only some clinical validation studies met the requirements for measurement of agreement. Clinical validation studies ideally need three different agreement measurements: Passing–Bablock/Deming regression analysis; Bland–Altman analysis displaying agreement; and assessment of predictive performance [24,107]. Only tacrolimus and mycophenolic acid were assessed with the three measurements and showed good agreement [61,108]. Hence, clinical validation of the other drugs, namely albendazole and radiprodil, must be completed with other agreement measurements that still need to be conducted [51,81].

## 5. Future Prospective of Volumetric Absorptive Microsampling (VAMS)

VAMS may be an alternative to CVS to support clinical applications, such as TDM and pharmacokinetic studies, for research purposes. There has been much research on developing and validating analytical methods for drugs taken with VAMS. Yet, in the validation process, the LLOQ concentration needs to be below clinically relevant concentrations of the drugs to ensure that the method can be appropriately applied. More research is needed on the clinical validation of VAMS to make this technique applicable in routine practice as an alternative or replacement for conventional sampling. During clinical validation studies, sample acquisition by well-trained personnel is critical in obtaining high-quality samples. Also, re-analysis of VAMS samples must be incorporated in future studies to prove the reproducibility of results; thus, the samples must be taken more than once.

## 6. Conclusions

VAMS is a promising sampling technique for TDM and pharmacokinetic studies, and assays of many drugs have been developed and analytically validated. However, only a few studies have performed a clinical validation study. All studies used Bland–Altman analysis to assess agreement; only tacrolimus and mycophenolic acid evaluated the performance to predict plasma concentrations from VAMS concentrations. Most studies showed good agreement between concentration measurement and the two matrices. Therefore, VAMS can replace CVS in routine practice, especially for tacrolimus and mycophenolic acid. On the other hand, further research needs to be conducted for other drugs, as clinical validation is required before VAMS can replace CVS in routine care.

## Figures and Tables

**Table 1:** Optimization and Validation of VAMS Assays for Acidic Drugs.

No	Drug	Extraction Solvent	Extraction Conditions	Additional Sample Preparation	Analytical Instrument	Validation Result		Ref.
						Available Parameter	Missing Parameter	
1	Fosfomycin	Methanol + IS	On a lateral shaker for 30 min at 1200 rpm	-	LC-MS/MS	Calibration range of 5–2000 µg/mL Linearity, accuracy and precision, sensitivity, selectivity, recovery, matrix effect, and carry-over were assessed and met the acceptance criteria Stability was only assessed for 500 µg/mL concentrations and was stable at RT for 10 days in an autosampler at 4 °C for 48 h	Stability for QC concentrations and dilution integrity was not assessed	[39]
2	Emixustat	Methanol containing 1% ammonium hydroxide + IS	Sonication for 15 min and vortex for 15 min	Evaporation at 40 °C with nitrogen, reconstitution in methanol–water (3:7, *v*/*v*) containing 0.1% formic acid	LC-MS/MS	Calibration range of 0.05–10 ng/mL Linearity, accuracy and precision, sensitivity, selectivity, recovery, matrix effect, dilution integrity, and carry-over were assessed and met the acceptance criteria Stable at RT for 1 week; −4 °C for 1 months; −20 °C for 1 months	-	[46]
3	Atorvastatin	Methanol–acetonitrile (1:1, *v*/*v*) and 1% formic acid + IS	Vortex for 1 h	Centrifugation at 3000 rpm for 15 min	LC-MS/MS	Calibration range of 1–2000 ng/mL Linearity, accuracy and precision, and recovery were assessed and met the acceptance criteria	Sensitivity, selectivity, matrix effect, dilution integrity, carry-over, and stability were not assessed	[13]
4	Tamoxifen	Methanol–acetonitrile (1:1, *v*/*v*) and 1% formic acid + IS	Vortex for 1 h	Centrifugation at 3000 rpm for 15 min	LC-MS/MS	Calibration range of 1–2000 ng/mL Linearity, accuracy and precision, and recovery were assessed and met the acceptance criteria	Sensitivity, selectivity, matrix effect, dilution integrity, carry-over, and stability were not assessed	[13]
5	Amiodarone	Methanol–acetonitrile (1:1, *v*/*v*) and 1% formic acid + IS	Vortex for 1 h	Centrifugation at 3000 rpm for 15 min	LC-MS/MS	Calibration range of 1–2000 ng/mL Linearity, accuracy and precision, and recovery were assessed and met the acceptance criteria	Sensitivity, selectivity, matrix effect, dilution integrity, carry-over, and stability were not assessed	[13]
6	Piperacillin	Methanol	Sonication for 10 min	Rehydration using water and incubation for 10 min at 37 °C in the thermos block heater	LC-MS/MS	Calibration range of 3.125–200 mg/L Linearity, accuracy and precision, sensitivity, selectivity, recovery, matrix effect, and carry-over were assessed and met the acceptance criteria Stable at RT and 4 °C for 72 h; −20 °C for 1 month	Dilution integrity was not assessed	[14]
7	Tazobactam	Methanol	Sonication for 10 min	Rehydration using water and incubation for 10 min at 37 °C in the thermos block heater	LC-MS/MS	Calibration range of 0.625–40 mg/L Linearity, accuracy and precision, sensitivity, selectivity, recovery, matrix effect, and carry-over were assessed and met the acceptance criteria Stable at RT and 4 °C for 72 h; −20 °C for 1 month	Dilution integrity was not assessed	[14]
8	Meropenem	Methanol	Sonication for 10 min	Rehydration using water and incubation for 10 min at 37 °C in the thermos block heater	LC-MS/MS	Calibration range of 0.625–40 mg/L Linearity, accuracy and precision, sensitivity, selectivity, recovery, matrix effect, and carry-over were assessed and met the acceptance criteria Stable at RT and 4 °C for 72 h; −20 °C for 1 month	Dilution integrity was not assessed	[14]
9	Ceftazidime	Methanol	Sonication for 10 min	Rehydration using water and incubation for 10 min at 37 °C in the thermos block heater	LC-MS/MS	Calibration range of 3.125–200 mg/L Linearity, accuracy and precision, sensitivity, selectivity, recovery, matrix effect, and carry-over were assessed and met the acceptance criteria Stable at 4 °C for 72 h; −20 °C for 1 month	Dilution integrity was not assessed	[14]
10	Paracetamol	Methanol–water–formic acid (80:20:0.01, *v*/*v*/*v*) + IS	Shaking for 15 min in a thermomixer comfort	Centrifugation at RT for 10 min at 10,000× *g*, supernatant dilution with water with 0.01% formic acid	LC-MS/MS	Calibration range of 4.0–32.8 µg/mL Linearity, accuracy and precision, sensitivity, selectivity, recovery, matrix effect, dilution integrity, and carry-over were assessed and met the acceptance criteria Stable at 50 °C for 1 week; −80 °C, −20 °C, and 4 °C for 9 months; RT for at least 8 months	-	[62]
11	Albendazole	Acetonitrile-methanol (1:1, *v*/*v*) with 0.1% formic acid + IS	Sonication in an ultrasonic bath for 1 h and agitation for 1 h in RT at 1200 rpm	Centrifugation for 10 min at 3300× *g*, evaporation of supernatant, and reconstitution in methanol	LC-MS/MS	Calibration range of 1–200 ng/mL Linearity, accuracy and precision, sensitivity, selectivity, recovery, and matrix effect were assessed and met the acceptance criteria Stable at RT for 2 months; in autosampler at 4 °C for 72 h; −80 °C for 2 months	Dilution integrity and carry-over were not assessed	[51]
12	Cefepime	Acetonitrile + IS	Sonication for 15 min, vortex at 700 rpm for 10 min, and centrifugation for 30 min at 3220× *g* at 4 °C	Rehydration using water, vortex for 2 min at 1000 rpm, and incubation for 10 min at 37 °C	LC-MS/MS	Calibration range of 0.100–100 µg/mL Linearity, accuracy and precision, sensitivity, selectivity, recovery, matrix effect, dilution integrity, and carry-over were assessed and met the acceptance criteria Stable at 4 °C for 1 week; −20 °C for 1 month (39 days); −78 °C for 3 months (91 days); in autosampler at 10 °C for 20 h	-	[64]
13	Ethosuximide	Acetonitrile + IS	Vortex mixing for 30 min at 600 rpm	Centrifugation for 10 min at 17,000× *g*, reconstitution of supernatant using water and 0.1% formic acid, and vortex for 30 s	LC-MS/MS	Calibration range of 3.48–234 mg/L Linearity, accuracy and precision, sensitivity, selectivity, recovery, matrix effect, dilution integrity, and carry-over were assessed and met the acceptance criteria Stable at −20 °C, 4 °C, 37 °C, 10 °C (autosampler) for 10 days; −20 °C for 4 months	-	[16]
14	Felbamate	Acetonitrile + IS	Vortex mixing for 30 min at 600 rpm	Centrifugation for 10 min at 17,000× *g*, reconstitution of supernatant using water and 0.1% formic acid, and vortex for 30 s	LC-MS/MS	Calibration range of 9.55–428 mg/L Linearity, accuracy and precision, sensitivity, selectivity, recovery, matrix effect, dilution integrity, and carry-over were assessed and met the acceptance criteria Stable at −20 °C, 4 °C, 37 °C, 10 °C (autosampler) for 10 days; −20 °C for 4 months	-	[16]
15	Phenobarbital	Acetonitrile + IS	Vortex mixing for 30 min at 600 rpm	Centrifugation for 10 min at 17,000× *g*, reconstitution of supernatant using water and 0.1% formic acid, and vortex for 30 s	LC-MS/MS	Calibration range of 0.89–60 mg/L Linearity, accuracy and precision, sensitivity, selectivity, recovery, matrix effect, dilution integrity, and carry-over were assessed and met the acceptance criteria Stable at −20 °C, 4 °C, 37 °C, 10 °C (autosampler) for 10 days; −20 °C for 4 months	-	[16]
16	Phenytoin	Acetonitrile + IS	Vortex mixing for 30 min at 600 rpm	Centrifugation for 10 min at 17,000× *g*, reconstitution of supernatant using water and 0.1% formic acid, and vortex for 30 s	LC-MS/MS	Calibration range of 0.58–39 mg/L Linearity, accuracy and precision, sensitivity, selectivity, recovery, matrix effect, dilution integrity, and carry-over were assessed and met the acceptance criteria Stable at −20 °C, 4 °C, 37 °C, 10 °C (autosampler) for 10 days; −20 °C for 4 months	-	[16]
17	Primidone	Acetonitrile + IS	Vortex mixing for 30 min at 600 rpm	Centrifugation for 10 min at 17,000× *g*, reconstitution of supernatant using water and 0.1% formic acid, and vortex for 30 s	LC-MS/MS	Calibration range of 0.76–51 mg/L Linearity, accuracy and precision, sensitivity, selectivity, recovery, matrix effect, dilution integrity, and carry-over were assessed and met the acceptance criteria Stable at −20 °C, 4 °C, 37 °C, 10 °C (autosampler) for 10 days; −20 °C for 4 months	-	[16]
18	Topiramate	Acetonitrile + IS	Vortex mixing for 30 min at 600 rpm	Centrifugation for 10 min at 17,000× *g*, reconstitution of supernatant using water and 0.1% formic acid, and vortex for 30 s	LC-MS/MS	Calibration range of 0.67–45 mg/L Linearity, accuracy and precision, sensitivity, selectivity, recovery, matrix effect, dilution integrity, and carry-over were assessed and met the acceptance criteria Stable at −20 °C, 4 °C, 37 °C, 10 °C (autosampler) for 10 days; −20 °C for 4 months	-	[16]
19	Zonisamide	Acetonitrile + IS	Vortex mixing for 30 min at 600 rpm	Centrifugation for 10 min at 17,000× *g*, reconstitution of supernatant using water and 0.1% formic acid, and vortex for 30 s	LC-MS/MS	Calibration range of 1.07–72 mg/L Linearity, accuracy and precision, sensitivity, selectivity, recovery, matrix effect, dilution integrity, and carry-over were assessed and met the acceptance criteria Stable at −20 °C, 4 °C, 37 °C, 10 °C (autosampler) for 10 days; −20 °C for 4 months	-	[16]
20	Mycophenolic acid	Water and methanol + IS	Sonication for 15 min	Centrifugation for 5 min at 14,500 rpm, evaporation of supernatant, and reconstitution using ammonium formate and 0.1% formic acid in water-acetonitrile (6:4, *v*/*v*)	LC-MS/MS	Calibration range of 75–7500 ng/mL Linearity, accuracy and precision, sensitivity, selectivity, recovery, matrix effect, and carry-over were assessed and met the acceptance criteria Stable in autosampler at 6 °C for 72 h; 20 °C, 4 °C, and RT for 15 days; −20 °C for 8 months	Dilution integrity was not assessed	[61]
21	Tamoxifen	Methanol + IS	Vortex for 1 min, sonication for 25 min	Dry in a water bath at 55 °C under nitrogen steam, reconstitution using 0.1% formic acid and 0.1% formic acid in acetonitrile, vortex for 20 s	LC-MS/MS	Calibration range of 2.5–200 ng/mL Linearity, accuracy and precision, sensitivity, selectivity, recovery, matrix effect, dilution integrity, and carry-over were assessed and met the acceptance criteria Stable at RT for 24 h; −20 °C for 1 month; in autosampler for 24 h	-	[63]

IS: internal standard; LC-MS/MS: liquid chromatograph–tandem mass spectrometer; RT: room temperature; QC: quality control.

**Table 2:** Optimization and Validation of VAMS Assays for Basic Drugs.

No	Drug	Extraction Solvent	Extraction Conditions	Additional Sample Preparation	Analytical Instrument	Validation Result		Ref.
						Available Parameter	Missing Parameter	
1	Paraxanthine	Methanol–water (8:2, *v*/*v*) and 0.01% formic acid + IS	In a thermomixer at 1000 rpm and 22 °C	Centrifugation at RT (10 min, 10,000× *g*), supernatant diluted with 0.01% formic acid	LC-MS/MS	Calibration range of 0.025–5 µg/mL Linearity, accuracy and precision, sensitivity, selectivity, recovery, matrix effect, and carry-over were assessed and met the acceptance criteria Stable at RT for 82 days; 60 °C for 4 days; −20 °C for 30 days; in autosampler at 4 °C for 4 days	Dilution integrity was not assessed	[28]
2	Caffeine	Methanol–water (8:2, *v*/*v*) and 0.01% formic acid + IS	In a thermomixer at 1000 rpm and 22 °C	Centrifugation at RT (10 min, 10,000× *g*), supernatant diluted with 0.01% formic acid	LC-MS/MS	Calibration range of 0.05–10 µg/mL Linearity, accuracy and precision, sensitivity, selectivity, recovery, matrix effect, and carry-over were assessed and met the acceptance criteria Stable at RT for 82 days; 60 °C for 4 days; −20 °C for 30 days; in autosampler at 4 °C for 4 days	Dilution integrity was not assessed	[28]
3	Miltefosine	Methanol	Mixing for 15 min at 1250 rpm and vortex for 30 s	A 20-fold dilution of highly concentrated samples with methanol and the addition of IS	LC-MS/MS	Calibration range of 10–5000 ng/mL Linearity, accuracy and precision, sensitivity, selectivity, recovery, matrix effect, dilution integrity, and carry-over were assessed and met the acceptance criteria Stable at RT for 1 month; 37 °C for 1 month; 2–8 °C for 5 days	-	[31]
4	Itraconazole	Acetonitrile-methanol (6:4, *v*/*v*) + IS	Incubation for 5 min, agitation for 5 min with a vortex mixer	Phospholipid removal with Ostroplate, evaporation under vacuum at 50 °C for 1 h, reconstitution in methanol–water (1:1, *v*/*v*)	LC-MS/MS	Calibration range of 10–1000 ng/mL Linearity, accuracy and precision, selectivity, recovery, and matrix effect were assessed and met the acceptance criteria Stable at RT for 24 h; −80 °C for 2 weeks; in autosampler at 6 °C for 24 h	Sensitivity, dilution integrity, and carry-over were not assessed	[48]
5	Cathinone analogs	Methanol	Ultrasound agitation for 15 min and vortex for 1 min	Evaporation under vacuum and reconstitution in acetonitrile-water (1:1, *v*/*v*) containing 0.1% formic acid	LC-MS/MS	Calibration range of 10–500 ng/mL Linearity, accuracy and precision, sensitivity, selectivity, recovery, and matrix effect were assessed and met the acceptance criteria Stable at RT for 7 days only for one concentration of 250 ng/mL	Dilution integrity, carry-over, and stability for QC concentrations were not assessed	[45]
6	Metoprolol	Methanol–acetonitrile (1:1, *v*/*v*) and 1% formic acid + IS	Vortex for 1 h	Centrifugation at 3000 rpm for 15 min	LC-MS/MS	Calibration range of 1–2000 ng/mL Linearity, accuracy and precision, and recovery were assessed and met the acceptance criteria	Sensitivity, selectivity, matrix effect, dilution integrity, carry-over, and stability were not assessed	[13]
7	Midazolam	Methanol–acetonitrile (1:1, *v*/*v*) and 1% formic acid + IS	Vortex for 1 h	Centrifugation at 3000 rpm for 15 min	LC-MS/MS	Calibration range of 1–2000 ng/mL Linearity, accuracy and precision, and recovery were assessed and met the acceptance criteria	Sensitivity, selectivity, matrix effect, dilution integrity, carry-over, and stability were not assessed	[13]
8	Salbutamol	Methanol + IS	Sonication for 15 min	Evaporation for 60 min, resuspended in N, O-Bis(trimethylsilyl)trifluoroacetamide (BSTFA) with 1% trimethylchlorosilane (TMCS) by vortexing, and derivatization at 60 °C for 30 min	GC-MS	Calibration range of 3–100 ng/mL Linearity, accuracy and precision, and recovery were assessed and met the acceptance criteria Stable at −20 °C, RT, and 30 °C for 145 days; 4 °C for 75 days	Sensitivity, selectivity, matrix effect, dilution integrity, and carry-over were not assessed	[78]
9	Hydroxychloroquine	Water + IS	Vortex for 30 min	Protein precipitation with 70% perchloric acid, centrifugation for 5 min at 14,000 rpm	LC-MS/MS	Calibration range of 10–2000 ng/mL Linearity, accuracy and precision, sensitivity, selectivity, recovery, matrix effect, dilution integrity, and carry-over were assessed and met the acceptance criteria Stable at RT for 10 days; 22 °C and 50 °C for 24 h	-	[38]
10	Linezolid	Methanol	Sonication for 10 min	Rehydration using water and incubation for 10 min at 37 °C in the thermos block heater	LC-MS/MS	Calibration range of 0.625–40 mg/L Linearity, accuracy and precision, sensitivity, selectivity, recovery, matrix effect, and carry-over were assessed and met the acceptance criteria Stable at RT and 4 °C for 72 h; −20 °C for 1 month	Dilution integrity was not assessed	[14]
11	Praziquantel	Acetonitrile-water (4:1, *v*/*v*) + IS	Thermos-mixing for 5 min at 1400 rpm at RT and ultrasonication for 40 min	-	LC-MS/MS	Calibration range of 0.2–50 µg/mL Linearity, accuracy and precision, sensitivity, recovery, and matrix effect were assessed and met the acceptance criteria Stable at RT for 4 months; 4 °C for 72 h; −80 °C for 2 months	Carry-over, selectivity, and dilution integrity were not assessed	[37]
12	Radiprodil	Methanol–ethyl acetate (1:1, *v*/*v*) + IS	Sonication for 15 min, shaking for 1 h at 1200 rpm	Dry using nitrogen and reconstitution	LC-ESI-MS/MS	Calibration range of 1–1000 ng/mL Linearity, accuracy, and precision were assessed and met the acceptance criteria	Sensitivity, selectivity, recovery, matrix effect, dilution integrity, carry-over, and stability were not assessed	[81]
13	Voriconazole	Acetonitrile-methanol (1:1, *v*/*v*) + IS	Sonication for 15 min, vortex for 30 min at 350 rpm	Centrifugation for 30 min at 4000 rpm	LC-MS/MS	Calibration range of 10–10,000 ng/mL Linearity, accuracy and precision, sensitivity, selectivity, recovery, matrix effect, dilution integrity, and carry-over were assessed and met the acceptance criteria Stable at RT for 19 h; −80 °C for 3 days; 4 °C, −20 °C, −80 °C for 1 month; in autosampler for 24 h	-	[80]
14	Voriconazole N-oxide	Acetonitrile-methanol (1:1, *v*/*v*) + IS	Sonication for 15 min, vortex for 30 min at 350 rpm	Centrifugation for 30 min at 4000 rpm	LC-MS/MS	Calibration range of 10–10,000 ng/mL Linearity, accuracy and precision, sensitivity, selectivity, recovery, matrix effect, dilution integrity, and carry-over were assessed and met the acceptance criteria Stable at RT for 19 h; −80 °C for 3 days; in autosampler for 24 h	-	[80]
15	Midazolam	Methanol + IS	On a lateral shaker for 1 h	-	LC-MS/MS	Calibration range of 5–5000 ng/mL Linearity, accuracy and precision, sensitivity, selectivity, recovery, matrix effect, and dilution integrity were assessed and met the acceptance criteria Stable at RT for 131 days; 4 °C for 192 h; 37 °C for 4 h; 40 °C for 43 days; −20 °C for 43 days	Carry-over was not assessed	[79]
16	Lamotrigine	Acetonitrile + IS	Vortex mixing for 30 min at 600 rpm	Centrifugation for 10 min at 17,000× *g*, reconstitution of supernatant using water and 0.1% formic acid, and vortex for 30 s	LC-MS/MS	Calibration range of 0.67–45 mg/L Linearity, accuracy and precision, sensitivity, selectivity, recovery, matrix effect, dilution integrity, and carry-over were assessed and met the acceptance criteria Stable at −20 °C, 4 °C, 37 °C, 10 °C (autosampler) for 10 days; −20 °C for 4 months	-	[16]
17	Rufinamide	Acetonitrile + IS	Vortex mixing for 30 min at 600 rpm	Centrifugation for 10 min at 17,000× *g*, reconstitution of supernatant using water and 0.1% formic acid, and vortex for 30 s	LC-MS/MS	Calibration range of 0.94–63 mg/L Linearity, accuracy and precision, sensitivity, selectivity, recovery, matrix effect, dilution integrity, and carry-over were assessed and met the acceptance criteria Stable at −20 °C, 4 °C, 37 °C, 10 °C (autosampler) for 10 days; −20 °C for 4 months	-	[16]
18	Mitotane	Methanol–aqueous zinc sulfate heptahydrate solution 2% (4:1, *v*/*v*)	Ultrasonic bath for 15 min, shaking for 1 h at 1400 rpm	Centrifugation at 12,000× *g* for 5 min at 4 °C	LC-UV	Calibration range of 1–50 mg/L Linearity, accuracy and precision, sensitivity, selectivity, recovery, and carry-over were assessed and met the acceptance criteria Stable at RT for 1 week; 2–8 °C for 1 week; in autosampler for 24 h	Matrix effect and dilution integrity were not assessed	[82]

IS: internal standard; LC-MS/MS: liquid chromatograph–tandem mass spectrometer; GC-MS: gas chromatograph–mass spectrometer; LC-ESI-MS/MS: liquid chromatograph–electrospray ionization–tandem mass spectrometer; LC-UV, liquid chromatograph–ultraviolet; RT, room temperature; QC, quality control.

**Table 3:** Optimization and Validation of VAMS Assays for Neutral Drugs.

No	Drug	Extraction Solvent	Extraction Conditions	Additional Sample Preparation	Analytical Instrument	Validation Result		Ref.
						Available Parameter	Missing Parameter	
1	Tacrolimus	Methanol–zinc sulfate 0.10 mol/L (2:1) + IS	Shaking on the orbital shaker at 1400 rpm for 6 min	Centrifugation at 2000× *g* for 10 min at 4 °C	LC-MS/MS	Calibration range of 0.7–6 µg/L Linearity, accuracy and precision, sensitivity, selectivity, recovery, matrix effect, dilution integrity, and carry-over were assessed and met the acceptance criteria Stable at RT for 2 months	-	[97]
2	Carbamazepine	Acetonitrile + IS	Vortex mixing for 30 min at 600 rpm	Centrifugation for 10 min at 17,000× *g*, reconstitution of supernatant using water and 0.1% formic acid, and vortex for 30 s	LC-MS/MS	Calibration range of 0.45–30 mg/L Linearity, accuracy and precision, sensitivity, selectivity, recovery, matrix effect, dilution integrity, and carry-over were assessed and met the acceptance criteria Stable at −20 °C, 4 °C, 37 °C, 10 °C (autosampler) for 10 days; −20 °C for 4 months	-	[16]
3	Lacosamide	Acetonitrile + IS	Vortex mixing for 30 min at 600 rpm	Centrifugation for 10 min at 17,000× *g*, reconstitution of supernatant using water and 0.1% formic acid, and vortex for 30 s	LC-MS/MS	Calibration range of 0.45–30 mg/L Linearity, accuracy and precision, sensitivity, selectivity, recovery, matrix effect, dilution integrity, and carry-over were assessed and met the acceptance criteria Stable at −20 °C, 4 °C, 37 °C, 10 °C (autosampler) for 10 days; −20 °C for 4 months	-	[16]
4	Oxcarbazepine	Acetonitrile + IS	Vortex mixing for 30 min at 600 rpm	Centrifugation for 10 min at 17,000× *g*, reconstitution of supernatant using water and 0.1% formic acid, and vortex for 30 s	LC-MS/MS	Calibration range of 0.18–12 mg/L Linearity, accuracy and precision, sensitivity, selectivity, recovery, matrix effect, dilution integrity, and carry-over were assessed and met the acceptance criteria Stable at −20 °C, 4 °C, 37 °C, 10 °C (autosampler) for 10 days; −20 °C for 4 months	-	[16]
5	Perampanel	Acetonitrile + IS	Vortex mixing for 30 min at 600 rpm	Centrifugation for 10 min at 17,000× *g*, reconstitution of supernatant using water and 0.1% formic acid, and vortex for 30 s	LC-MS/MS	Calibration range of 0.03–1.86 mg/L Linearity, accuracy and precision, sensitivity, selectivity, recovery, matrix effect, dilution integrity, and carry-over were assessed and met the acceptance criteria Stable at −20 °C, 4 °C, 37 °C, 10 °C (autosampler) for 10 days; −20 °C for 4 months	-	[16]
6	Levetiracetam	Acetonitrile + IS	Vortex mixing for 30 min at 600 rpm	Centrifugation for 10 min at 17,000× *g*, reconstitution of supernatant using water and 0.1% formic acid, and vortex for 30 s	LC-MS/MS	Calibration range of 0.94–63 mg/L Linearity, accuracy and precision, sensitivity, selectivity, recovery, matrix effect, dilution integrity, and carry-over were assessed and met the acceptance criteria Stable at −20 °C, 4 °C, 37 °C, 10 °C (autosampler) for 10 days; −20 °C for 4 months	-	[16]
7	Tacrolimus	Water and methanol + IS	Sonication for 15 min	Centrifugation for 5 min at 14,500 rpm, evaporation of supernatant, and reconstitution using ammonium formate and 0.1% formic acid in water-acetonitrile (6:4, *v*/*v*)	LC-MS/MS	Calibration range of 0.5–50 ng/mL Linearity, accuracy and precision, sensitivity, selectivity, recovery, matrix effect, and carry-over were assessed and met the acceptance criteria Stable in autosampler at 6 °C for 72 h; 20 °C, 4 °C, and RT for 15 days; −20 °C for 8 months	Dilution integrity was not assessed	[61]
8	Sirolimus	Water and methanol + IS	Sonication for 15 min	Centrifugation for 5 min at 14,500 rpm, evaporation of supernatant, and reconstitution using ammonium formate and 0.1% formic acid in water-acetonitrile (6:4, *v*/*v*)	LC-MS/MS	Calibration range of 0.5–50 ng/mL Linearity, accuracy and precision, sensitivity, selectivity, recovery, matrix effect, and carry-over were assessed and met the acceptance criteria Stable in autosampler at 6 °C for 72 h; 20 °C, 4 °C, and RT for 15 days; −20 °C for 8 months	Dilution integrity was not assessed	[61]
9	Everolimus	Water and methanol + IS	Sonication for 15 min	Centrifugation for 5 min at 14,500 rpm, evaporation of supernatant, and reconstitution using ⁰ammonium formate and 0.1% formic acid in water-acetonitrile (6:4, *v*/*v*)	LC-MS/MS	Calibration range of 0.5–50 ng/mL Linearity, accuracy and precision, sensitivity, selectivity, recovery, matrix effect, and carry-over were assessed and met the acceptance criteria Stable in autosampler at 6 °C for 72 h; 20 °C, 4 °C, and RT for 15 days; −20 °C for 8 months	Dilution integrity was not assessed	[61]
10	Cyclosporin A	Water and methanol + IS	Sonication for 15 min	Centrifugation for 5 min at 14,500 rpm, evaporation of supernatant, and reconstitution using ammonium formate and 0.1% formic acid in water-acetonitrile (6:4, *v*/*v*)	LC-MS/MS	Calibration range of 20–2000 ng/mL Linearity, accuracy and precision, sensitivity, selectivity, recovery, matrix effect, and carry-over were assessed and met the acceptance criteria Stable in autosampler at 6 °C for 72 h; 20 °C, 4 °C, and RT for 15 days; −20 °C for 8 months	Dilution integrity was not assessed	[61]
11	Tacrolimus	Acetonitrile-zinc sulfate 0.05 M in water (1:1, *v*/*v*) + IS	Vortex for 1 min	Addition of ammonium sulfate 40% for salting out, vortex for 1 min, centrifugation at 16,260× *g* for 5 min at 8 °C	LC-MS/MS	Calibration range of 2.25–42.9 ng/mL Linearity, accuracy and precision, sensitivity, selectivity, recovery, matrix effect, and carry-over were assessed and met the acceptance criteria Stable at RT for 7 days; 4 °C and 60 °C for 48 h; −80 °C for 1 month	Dilution integrity was not assessed	[53]

IS: internal standard; LC-MS/MS: liquid chromatograph–tandem mass spectrometer; RT: room temperature.

**Table 4:** Clinical Validation of VAMS Assays versus Assays for CVS for Several Drugs.

No	Drug	Sample (*n*)	Patient (*n*)	Measures of Clinical Validation			Ref.
				Passing–Bablock Analysis (Slope [95% CI])	Bland–Altman Analysis	Predictive Performance	
1	Albendazole	100	10	NA	Good Agreement	NA	[51]
2	Tacrolimus	88	72	Linear (0.88 [0.81; 0.97]) ^a^ (1.00 [0.98; 1.02]) ^b^	Good agreement (after correction)	Met the acceptance criteria	[108]
		679	27	Linear (1.01 [NA])	Good agreement	NA	[97]
		53	53	Linear (1.26 [1.16; 1.4]) ^a^ (0.99 [0.91; 1.07]) ^b^	Good agreement (after correction)	Met the acceptance criteria	[61]
		97	25	Linear (1.05 [0.98; 1.14])	Good agreement	NA	[109]
3	Radiprodil	150	10	NA	Good agreement	NA	[81]
4	Mycophenolic acid	20	20	Linear (0.98 [0.94; 1.05])	Good agreement	Met the acceptance criteria	[61]
		64	25	Linear (0.72 [0.66; 0.77]) ^a^ (1.07 [0.97; 1.13]) ^b^	Good agreement (after correction)	NA	[109]

NA, not available; ^a^, uncorrected concentration; ^b^, corrected concentration.

## Data Availability

Data sharing not applicable.
